# Impact of mass media on the utilization of antenatal care services among women of rural community in Nepal

**DOI:** 10.1186/s13104-015-1312-8

**Published:** 2015-08-12

**Authors:** Dilaram Acharya, Vishnu Khanal, Jitendra Kumar Singh, Mandira Adhikari, Salila Gautam

**Affiliations:** Department of Public Health, Purbanchal University, Sanjeevani College of Medical Science, Rupandehi, Nepal; School of Public Health, Curtin University, Perth, Australia; Department of Community Medicine, Tribhuwan University, Janaki Medical College, Janakpur, Nepal; Population Services International, Nawalparasi, Nepal; Nepal Development Society, Bharatpur, Nepal

**Keywords:** Antenatal care, Impact, Mass media, Nepal

## Abstract

**Background:**

Antenatal care has several benefits for expecting mothers and birth outcomes; yet many mothers do not utilise this service in Nepal. Mass media may play an important role in increasing the use of antenatal care and other maternal health services. However, the effect of mass media on increasing health service utilisation has remained an under studied area in Nepal. The aim of this study was to investigate the impact of mass media on the utilisation of antenatal care services in rural Nepal.

**Methods:**

A community-based cross-sectional study was conducted in Sinurjoda Village Development Committee of Dhanusha District, Nepal. A total of 205 mothers of children aged under 1 year were selected using systematic random sampling. Logistic regression was employed to examine the association between selected antenatal care services and mass media exposure after adjusting for other independent variables.

**Results:**

A majority of mothers were exposed to mass media. Radio was accessible to most (60.0 %) of the participants followed by television (43.41 %). Mothers exposed to mass media were more likely to attending antenatal visits [Odds ratio (OR) 6.28; 95 % CI (1.01–38.99)], taking rest and sleep during pregnancy [OR 2.65; 95 % CI (1.13–6.26)], and receiving TT immunization [OR 5.12; 95 % CI (1.23–21.24)] than their non-exposed counterparts.

**Conclusions:**

The study reported a positive influence of mass media on the utilisation of antenatal care services in Nepal. Therefore, further emphasis should be given to increase awareness of women of rural Nepal through mass media to improve utilisation of antenatal care services in Nepal.

## Background

Worldwide 292,982 women were lost their lives in 2013 [1]. Of all maternal deaths, about 87 % of maternal deaths occur in Sub-Saharan Africa and South Asia showing that developing countries share an unequal burden of maternal deaths [2]. Nepal has a high maternal mortality (229 per 100,000 live births) which is likely to be reduced with appropriate intervention [3].

Access to timely and appropriate health care during pregnancy and childbirth is the most effective health interventions for safer and healthier outcome for maternal and newborn survival [4, 5]. Antenatal care (ANC) is a key strategy to reducing maternal and neonatal morbidity and mortality [6, 7]. The aim of ANC is detection and treatment of complications; and most importantly the promotion of maternal nutrition, rest and care during the period to improve pregnancy outcome [8]. Increased ANC coverage has also been reported to be effective in increasing skilled attendance at delivery especially delivery in health facilities which have been found to be effective in reducing maternal mortality in developing countries [9, 10]. While the benefits of attending ANC are widely acknowledged, millions of women in developing countries are not receiving such care as per recommended standard of frequency and quality [6].

To increase ANC attendance, reaching mothers, their families and community opinion leaders is essential so that they understand the importance of ANC visits. Mass media has been shown to be an effective measure to reach mothers in a large scale and increase the use of maternal health services, especially in developing countries. For example, women who read newspapers (OR: 2.92, 95 % CI: 1.15, 7.40), or reported watching television (OR: 3.06; 95 % CI: 1.70, 5.51) in Bangladesh were almost three times more likely to attend ANC [8]. Studies from India [11] and Uganda [12] has also found positive effect of mass media on increasing ANC attendance.

In general, during ANC, mothers receive check-up, immunization against tetanus toxoid, consumption of all recommended iron folic acid (IFA) tablets and syrup [13]. There is lack of literature on the impact of media exposure on the utilisation of ANC in Nepal. A previous study from Nepal reported that age, education, parity and wealth were associated with both the timing and the frequency of ANC visits, however, the study also paid no particular attention to exposure to mass media and its impact on attendance [14]. More recently, Joshi et al. [13] also reported on factors associated with ANC in Nepal; however, similar to previous study, they did not assess the potential impact of mass media on ANC utilisation.

In the current study setting, where 47 % of women attend four ANC, and 32 % are literate [15], mass media such as radio and television may be effective means to disseminating message to increase the utilisation of maternal health services. However, there is lack of evidence to support this argument. Increasing evidence on such factors would enable policy makers to design evidence based communication program to improve the uptake of ANC and other maternal health services. This study aimed to examine the impact of mass media exposure to the ANC service utilisation.

## Methods

### Study design and sampling

#### Study setting

Data were collected between March 2012 and May 2012 in Sinurjoda Village Development Committee (VDC) of Dhanusha District, Nepal. The study area is located in Eastern Development Region of Nepal. Village Development Committee (VDC) is the lowest administrative unit of rural areas of Nepal. This VDC has a population of 9,457 (male: 4,396; female: 5,061). The total literacy rate of district was 65 % with female literacy rate only 32 % [15]. ANC attendance has remained low in the district with 76 % of women attending at least one ANC visit and 47 % attending the recommended four or more. Skilled attendance at birth is still much lower (13 %) than the national average of 35.3 % [15, 16].

#### Study design and sampling

For this community based cross sectional study, mother with infants aged under 1 year infants were selected using systematic random sampling. Of the total 3,416 households; 1,007 households had the children less than 1 year of age according to the district health office annual target. Sample size for the study was calculated with the standard formula 4pq/L^2^. Where, ‘p’ was assumed 0.15 taking into account from Nepal Demographic and Health Survey (NDHS) 2011 [16], which shows that 15 % of women in rural Nepal having access to “Radio Nepal (the national radio)” as media source for health-related information, q = 0.85 (non access to radio Nepal) and L was taken 5 % (assumed) as allowable error. This yielded a total sample size of 204. The final sample size for this study was 205.

### Data collection

The structured questionnaire was administered using face-to-face interviews. The English version of the questionnaire was adapted from the Nepal Demographic and Health Survey [16] and were translated to Nepali and back translated to English to ensure no variation in the meaning of the questions. The Nepali version of questionnaire was pretested in a neighbouring VDC on a similar group of mothers to ensure cultural adaptability. The questionnaire consisted of three parts: (1) respondent’s exposure to specific mass media at least once a week; (2) selected background characteristics of the respondents; and (3) the utilisation of ANC. Five medical students, who were trained on questionnaire administration, conducted the interviews.

### Ethics

The research proposal was approved by the Ethical Committee for Health Research of Janaki Medical College Teaching Hospital, Tribhuvan University, Nepal. Mothers provided consent before interview. Personal identifiers were removed before data analysis.

### Definition of variables

The outcome variable of the study was the utilization of antenatal care components including frequency of ANC, nutritional supplementation (additional food intake, and iron folic acid supplementation intake), de-worming, Tetanus Toxoid (TT) immunization, rest and sleep, physical and laboratory examination, and ANC provider (skilled vs. unskilled).

The explanatory variable of interest of this study was exposure to mass media. Other independent variables in the study were: age, caste/ethnicity, educational status, occupation and the standard of living.

Mothers’ age was coded as <20 years, 20–35 years and >35 years. Ethnicity was based on the caste system in Nepal and was divided into three major groups based on available literature and similarities between the caste/ethnic groups: (1) advantaged (2) disadvantaged (Janjati) (3) disadvantaged (Dalit) [17]. The advantaged groups were non-dalit terai caste and upper caste groups [17]. Maternal education was recorded as no education, primary, some secondary, school leaving certificate (grade 10) and above. Maternal occupation was coded as working in agriculture, business/service, and labor (skilled and unskilled).

Standard of Living Index (SLI) was calculated on the basis of house type, availability of electricity, fuel used for cooking, presence of livestock and possession of household items including motorbike, cycle, television, radio, fan. A score as assigned for each factor and the sum of score for all the factors was taken as SLI. On the basis of total score, SLI was classified into three categories: low (less than nine), medium (9–20), and high (more than 20).

### Statistical analysis

The main exposure variable of interest was divided into two groups: exposed to mass media (reads newspaper, watches television, listens to radio, and has contact with health workers) at least once a week (n = 122); and non-exposed [having none of them (n = 83)]. The association between the independent variables and the use of antenatal care components were examined using Chi-square test. Significant outcome variables were then analysed using stepwise backward elimination in multiple logistic regression. Each ANC components were treated as outcome variables while examining such association after adjusting for other factors in the model. A P-value ≤0.05 was considered statistically significant. Data were analysed using Statistical Package for Social Sciences (SPSS version 17 for windows).

## Results

Table 1 shows the percentage of respondents exposed to specific mass media at least once a week. More than half (60.0 %) of the respondents were exposed to radio followed by television (43.1 %), printed materials (29.7), health worker (28.8 %), newspapers (16.5 %).

**Table 1:** Exposure of mass media as a source of information about health

Mass media	Number^a^	Percentage
Radio	122	59.52
Television	89	43.41
Printed materials	61	29.75
Wall paintings	58	28.29
Newspaper	34	16.58
Health workers	59	28.78
None	83	40.48

^a^Multiple response; recorded as at least once a week.

Table 2 presents the exposure of mass media in relation to socio-demographic characteristics of the respondents. The majority of mothers (64.8 %) were from 20 to 35 years age group. Less than half of the mothers (44.4 %) were illiterate. More than three-fourths of the respondents were involved in agriculture.

**Table 2:** Characteristics of the respondents among exposed and not exposed to mass media within a week

Characteristics	Total number (%)	Exposed (n = 122)	Not exposed (n = 83)	P value*
Age (in years)				
<20	46 (22.43)	25 (20.5)	21 (25.3)	0.174
20–35	133 (64.8)	85 (69.7)	48 (57.8)	
>35	26 (12.68)	12 (9.8)	14 (16.9)	
Caste/ethnicity				
Dalit	43 (20.97)	12 (9.8)	31 (37.3)	<0.001
Janjati	22 (10.73)	9 (7.4)	13 (15.7)	
Non Dalit Tarai caste	87 (42.43)	60 (49.2)	27 (32.5)	
Upper caste group	53 (25.85)	41 (33.6)	12 (14.5)	
Educational status				
No education	91 (44.39)	37 (30.3)	54 (65.1)	<0.001
Primary	40 (19.51)	28 (23.0)	12 (14.5)	
Some secondary	47 (22.92)	36 (29.5)	11 (13.3)	
SLC and above	27 (13.17)	21 (17.2)	6 (7.2)	
Occupation				
Agriculture	161 (78.53)	97 (79.5)	64 (77.1)	0.107
Business/service	24 (11.70)	17 (13.9)	7 (8.4)	
Labor	20 (9.75)	8 (6.6)	12 (14.5)	
Standard of Living Index (SLI)				
Low	41 (20)	15 (12.3)	26 (31.3)	<0.001
Middle	131 (63.90)	92 (75.4)	39 (47.0)	
High	33 (16.09)	15 (12.3)	18 (21.7)	
Total	205	122	83	

* P-value from Chi-square test

A number of components of antenatal care such as frequency of antenatal visits, nutritional supplementation, and physical examination, TT immunization, rest and sleep were significantly associated with exposure to mass media by univariate analysis (Table 3). In addition, maternal education (OR: 1.93; 95 % CI: 1.44, 2.59), and SLI (OR: 4.08; 95 % CI: 1.95, 8.55) were also significantly associated with the utilisation of antenatal care in unadjusted analyses. On the other hand, occupation was not significant (OR: OR 0.78; 95 % CI: 0.5, 1.2) (Not shown in table).

**Table 3:** Utilization of selected antenatal care services among participants exposed and unexposed to mass media within a week (unadjusted odds ratio)

Services	Exposed n./total (%) (n = 122)	Not exposed n./total (%) (n = 83)	OR (95 % CI)	P value
ANC Visit (yes vs. no)	98/122 (80.3)	40/83 (48.2)	4.39 (2.36–8.16)	<0.01
Freq. of ANC (≥4 vs. <4)	38/98 (38.77)	7/40 (17.5)	2.98 (1.2–7.4)	0.016
Nutritional supplementation and de-worming				
Additional food consumed	104/122 (85.2)	57/83 (68.7)	2.63 (1.33–5.21)	0.005
Iron and folic acid taken	93/98 (94.89)	31/40 (77.5)	5.40 (1.68–17.33)	0.002
De-worming	32/98 (32.65)	12/40 (30.0)	1.13 (0.51–2.51)	0.762
TT immunization	106/122 (86.9)	46/83 (55.4)	5.32 (2.69–10.52)	<0.001
Rest and sleep	65/122 (53.3)	23/83 (27.7)	2.97 (1.63–5.40)	<0.001
Physical and laboratory examination				
Weight measured	72/98 (73.46)	27/40 (67.5)	1.84 (0.87–3.89)	0.48
Blood pressure measured	72/98 (73.46)	19/40 (47.5)	3.06 (1.42–6.58)	0.003
Urine sample tested	60/98 (61.22)	22/40 (55.5)	2.38 (0.90–6.28)	0.499
Blood sample tested	66/98 (67.34)	25/40 (62.5)	1.23 (0.57–2.66)	0.586
Ultrasonography done	47/98 (47.95)	10/40 (25.0)	2.76 (1.22–6.26)	0.013
ANC provider (skilled vs. unskilled)^a^	82/98 (63.67)	25/40 (62.5)	3.07 (1.33–7.08)	0.007
Total	122	83		

* n.* frequency.

^a^Skilled Health Personnel: Doctor/HA/AHW/ANM/Nurse. Chi square test of association.

The final results of multiple logistic regression analyses are shown in Table 4. Mothers exposed to mass media were more likely to be attending ANC visit [OR 6.28; 95 % CI (1.01, 38.99)], rest and sleep during pregnancy [OR 2.65; 95 % CI (1.13, 6.26, P = 0.025)], and TT immunization [OR 5.12; 95 % CI (1.23, 21.24)] were significantly associated with the exposure to mass media. Maternal education, occupation and SLI were not significant in final model.

**Table 4:** Effect of mass media exposure on the utilisation of ANC using logistic regression model

Variables	Regression coefficient	SE	OR	95 % CI	P value
ANC visit (yes vs. no)	1.837	0.932	6.28	1.01–38.99	0.049
Rest and sleep	0.976	0.434	2.65	1.13–6.21	0.025
TT immunization	1.633	0.726	5.12	1.23–21.24	0.024

Variables entered for ANC visits (yes vs no), rest and sleep, TT immunization, education, occupation and SLI.

## Discussion

Our study showed that 60 and 43.1 % of the respondents had exposure to radio and television at least once a week, respectively. These figures are relatively high compared to national figures collected by the 2011NDHS, where around the country 44 % of women are estimated to listen to the radio, and 47 % watch television at least once a week [16]. Such an increase in the exposure status can be attributable to growing numbers of radio stations and television channels which have been recently established in the plain/Terai corridor of Nepal.

We found that mass media had positive impact on the utilisation of various ANC components such as frequency of antenatal visits, nutritional supplementation, physical examination, TT immunization, rest and sleep. The current findings accord with other studies from Bangladesh and India where exposure to mass media had positive influence in increasing antenatal care visits [11, 18, 19]. A Ugandan study also reported that women having access to media were more likely to use antenatal care compared to those with no access [12].

It should be noted that the majority of mothers in our study setting receive ANC in rural health facilities such as health post, sub health posts and also from outreach clinics [16]. In such setting, it is hard to reach all mothers by health workers and therefore, only mass media remains a viable option to reaching families and communities to disseminate message on the importance of ANC attendance and availability of such service in their own communities.

This study has provided evidence that mass media have positive effect on the utilisation of maternal health services. To the best of authors’ knowledge, no previous study has been reported on the influence of mass media on ANC from Southern Nepal. Nevertheless, a number of factors need to be considered as the limitation of this study. First, this was cross-sectional study which limits cause-effect relationship of mass media and service utilisation. Second, this study was confined to only one village development committee of the district which limits the generalizability of findings. Nevertheless, the current findings provide support for positive influence of mass media on health service utilisation in Nepal. Further intervention studies may be useful to examine the effects of different media and messages to increase the use of maternal health services in Nepal.

## Conclusion

Utilisation of ANC services was associated with exposure to mass media. Mothers exposed to mass media were more likely to be taking ANC visit, rest and sleep, and receive TT immunization during pregnancy. These findings suggest that for rural settings, utilising mass media to disseminate ANC messages may be an effective approach to improve the uptake of maternal health services and maternal and child survival in Nepal.
